# The *vaa *locus of *Mycoplasma hominis *contains a divergent genetic islet encoding a putative membrane protein

**DOI:** 10.1186/1471-2180-4-37

**Published:** 2004-09-22

**Authors:** Thomas Boesen, Jeppe Emmersen, Agata Baczynska, Svend Birkelund, Gunna Christiansen

**Affiliations:** 1 Department of Medical Microbiology and Immunology, University of Aarhus, DK-8000 Aarhus C, Denmark; 2 Department of Molecular Biology, Science Park, University of Aarhus, Gustav Wieds Vej 10C, DK-8000 Aarhus C, Denmark; 3 Department of Biotechnology, Aalborg University, DK-9000 Aalborg, Denmark

## Abstract

**Background:**

The *Mycoplasma hominis vaa *gene encodes a highly variable, surface antigen involved in the adhesion to host cells. We have analysed the structure of the *vaa *locus to elucidate the genetic basis for variation of *vaa*.

**Results:**

Mapping of *vaa *on existing physical maps of five *M. hominis *isolates by pulsed field gel electrophoresis revealed that *vaa *is located in a genomic region containing the majority of other characterized membrane protein genes of *M. hominis*. Sequencing of an 11 kb region containing the *vaa *locus of *M. hominis *isolate 132 showed the presence of conserved housekeeping genes at the borders of the region, *uvrA *upstream and the *hitABL *operon downstream to *vaa*. Analysis of 20 *M. hominis *isolates revealed that the *vaa *upstream region was conserved whereas the downstream region was highly variable. In isolate 132 this region contained an open reading frame (ORF) encoding a putative 160 kDa membrane protein. Homologous ORFs were present in half of the isolates, whereas this ORF, termed *vmp *(variable membrane protein), was deleted from the locus in the remaining isolates. Compellingly, the conserved upstream region and variable downstream region of *vaa *correlates with the genetic structure of *vaa *itself which consists of a conserved 5' end and a variable 3' end containing a variable number of exchangeable sequence cassettes.

**Conclusion:**

Our data demonstrate that the *vaa *locus contains a divergent genetic islet, and indicate pronounced intraspecies recombination. The high variability level of the locus indicate that it is a chromosomal 'hot spot', presumably important for sustaining diversity and a high adaptation potential of *M. hominis*.

## Background

The mycoplasmas are wall-less prokaryotes characterized by small genomes (580 – 2200 kb) and a low G+C content, generally below 30%. They are the smallest self-replicating organisms known with cell diameters normally in the range of 0.3–0.8 μm [1], and are observed as parasites of insects, plants, animals and humans with strict host specificities. As a consequence of the direct exposure of proteins located on the surface of the cytoplasmic membrane to the surrounding environment, antigenic variation of surface proteins is observed among mycoplasmas. The often chronic nature of mycoplasmal infections is thought to be a consequence of evasion of the humoral immune response by the variation displayed by these coat proteins [2].

*Mycoplasma hominis *is an opportunistic human pathogen observed as a commensal of the urogenital tract. Primarily, urogenital infections giving rise to spontaneous abortions, pelvic inflammatory disease, and acute pyelonephritis have been associated with *M. hominis*, but extragenital infections resulting in infant meningitis, arthritis, and septicemia have been reported [3].

*M. hominis *is a very heterogeneous species as measured by a pronounced antigenic variation [4-7]. The molecular basis for antigen variation of *M. hominis *surface proteins has been elucidated in some cases. The large membrane protein (*lmp*) gene family displays size variation by insertion/deletion of intragenic repeats of approximately 500 bp. The *lmp *genes are arranged in two clusters, *lmp1-2 *and *lmp3-4 *in the *M. hominis *genome of most analysed isolates with a distance between the clusters of more than 110 kb [8]. At least one member of the *lmp *family is expressed in each of the *M. hominis *isolates tested and decrease in the number of repeats were found to correlate to the amount of spontaneous agglutination of *M. hominis *cells [9,10].

The *vaa *(variable adherence-associated) gene encodes a size and phase variable *M. hominis *adhesin [11-14]. Phase variation is accomplished by variation in the number of bases in a poly-A tract situated in the 5'-end of the gene [12]. This is presumably achieved by slipped strand mispairing with a frequency of 10^-3^–10^-4^. In the ON-state 8 adenines are observed in the poly-A tract, whereas 7 or 9 results in the out-of-frame OFF-state [12]. A single *vaa *gene is present in each *M. hominis *isolate [15]. The size of Vaa observed in different isolates ranges from 28 kDa to 72 kDa. This size variation is the consequence of a variable number of homologous, exchangeable cassette sequences located in the 3' end of *vaa *[11,13]. Each cassette encodes approximately 110 aa containing a coiled-coil motif and 1 to 5 cassettes have been observed in different Vaa proteins. The Vaa protein is a rod-shaped, monomeric protein and the cassettes are presumed to form homologous, 'spike'-formed binding domains arranged in parallel in the three-dimensional structure [16]. Based on the cassette composition, 6 distinct *vaa *gene types have been observed in more than 100 analysed clinical isolates [13,14]. The mechanism behind this variation is unknown, but duplication/deletion of cassettes followed by divergence of cassette sequences has been suggested [14]. Comparison of the homology between cassettes showed that the cassette sequences could be divided arbitrarily into 5 cassette types based on sequence identity. Analysis of the cassette organization in different isolates revealed that intraspecies recombination resulting in the exchange of cassette sequences could be an alternative mechanism for variation of Vaa [13].

To obtain a better understanding of the genomic basis for variation of *vaa *and variation mechanisms in *M. hominis *in general, the *vaa *locus was characterized by mapping of the genomic position in five isolates and sequencing of a 11 kb region containing the *vaa *gene from isolate 132. Furthermore, the *vaa *locus of 20 *M. hominis *isolates was investigated by PCR and sequencing. In contrast to the more conserved *vaa *upstream region this analysis revealed that the downstream region also exhibits major variation caused by insertion/deletion and sequence variation of a large ORF encoding a putative membrane protein. Thus the *vaa *locus seems to constitute a 'hot spot' for variation in the *M. hominis *genome.

## Results

### Genomic localization of the *vaa *gene

**Figure 1:**
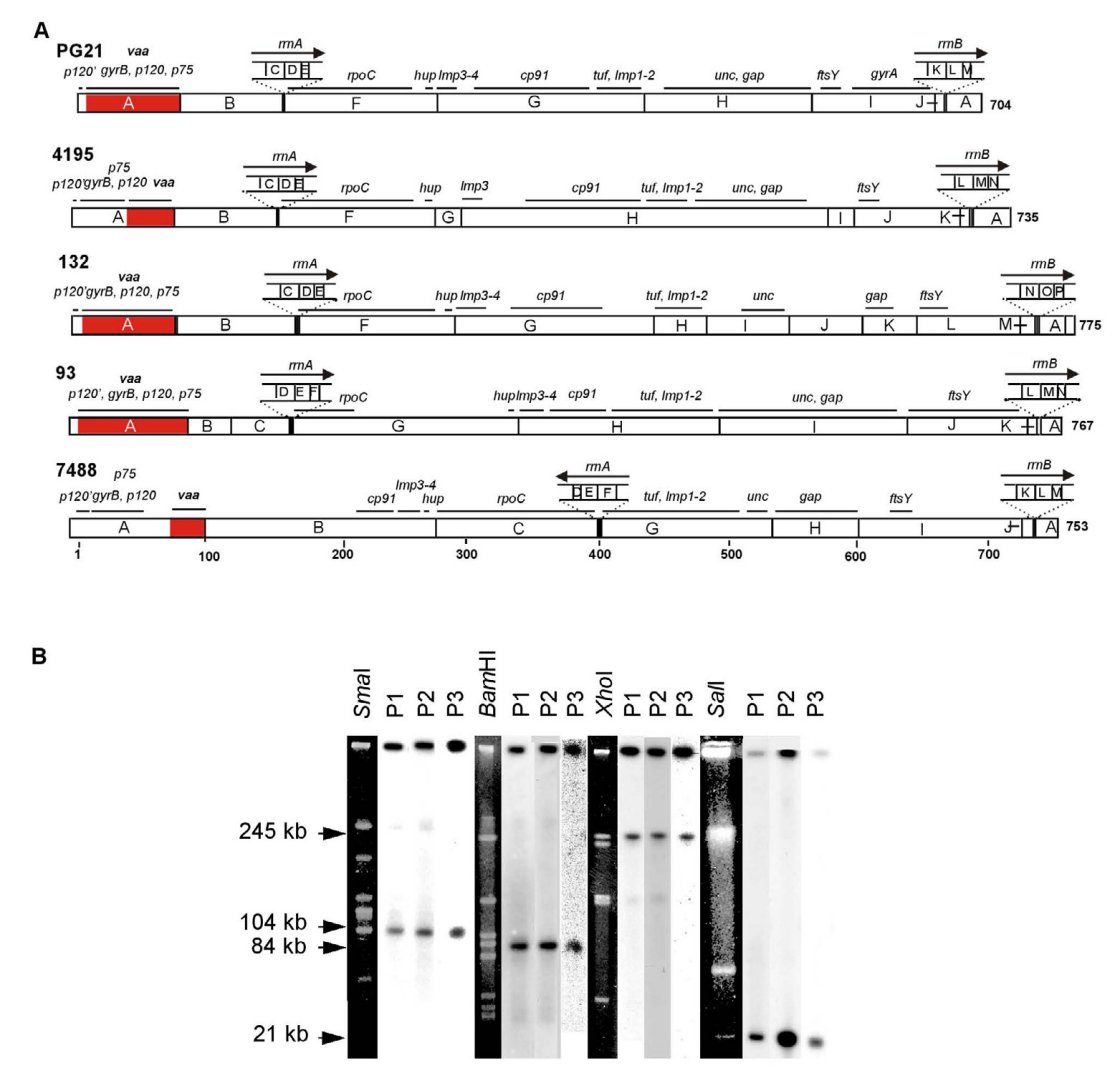
**Mapping the *vaa *gene. **The *vaa *gene was mapped on existing physical and genetic maps using pulsed field gel electrophoresis and Southern blotting [8]. A: the location of the *vaa *gene on the physical maps of five *M. hominis *isolates PG21, 4195, 132, 93 and 7488. The *vaa *gene mapped to the same genomic region for all isolates (shown in red). B: Pulsed field gel electrophoresis and Southern blotting results used for mapping of *vaa *in *M. hominis *7488. Digests of four out of five enzymes used is shown. The probes 1 to 3 are denoted P1 to 3. The sizes of the bands reacting with the probes are indicated on the left side of the figure.

The *vaa *gene was mapped on exsisting physical and genetic maps of 5 *M. hominis *isolates: 132, 4195, 7488, PG21, and 93 (Fig. 1A) [8]. Chromosomal DNA from the isolates was digested by the restriction endonucleases *Sma*I, *Bam*HI, *Xho*I, *Sal*I, and *Apa*I and the fragments were separated by pulsed field gel electrophoresis (Fig. 1B). The fragments were transferred to nitrocellulose and hybridized with three (α-^32^P)dATP-labelled DNA fragments representing different parts of selected *vaa *genes (see materials and methods). All probes hybridized to a single fragment in all digests, corresponding to the same region of the genome for all 5 isolates (Fig. 1A and 1B). The fragments were located in a genomic region near the *gyrB *gene (Fig. 1A). A number of other *M. hominis *genes encoding membrane proteins (*p75*, *p120 *and *p120'*, see Fig. 1A) were also positioned in this region of the genome [8,17,18].

**Figure 2:**
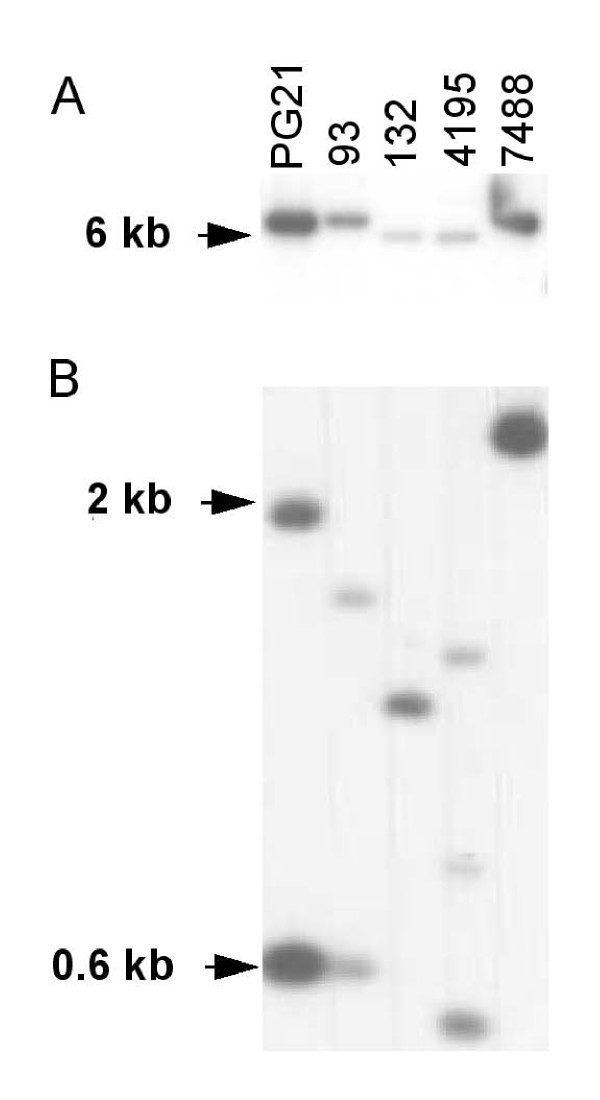
**Restriction endonuclease analysis (REA) of the *vaa *locus. **Southern blottings of *Hin*dIII cleaved genomic DNA of the 5 isolates separated by agarose gel electrophoresis and probed with sequences specific for the 5' end (probe 2, frame A) and 3' end (probe 4, frame B) of *vaa*, respectively.

To further analyse the variability of the vaa locus, restriction endonuclease analysis was performed. Southern blotting of *Hin*dIII and *Eco*RI cleaved genomic DNA separated with ordinary agarose gel electrophoresis from the 5 isolates using probe 2 was made (Fig. 2A and 2B). The probe recognized a band of 6.0 kb in the isolates 132 and 4195 and a band of 6.3 kb in isolates 7488, PG21, and 93 in the *Hin*dIII digest (Fig. 2A). The band size difference between the isolates could be attributed to the fact that the *Hin*dIII site relative to the ATG start codon in the *vaa *genes of isolates 7488, PG21, and 93 was located 243 bp further upstream compared to the *Hin*dIII site in isolates 132 and 4195. This indicates that the *Hin*dIII site upstream *vaa *is conserved. Bands of diverging size were observed in the isolates for the *Eco*RI digest using this probe (data not shown). The band differences observed could be explained by the presence or absence of an *Eco*RI site in the variable *vaa *gene. Using a probe comprising the cassette region of the *vaa *category 3 (*vaa-*3) gene (probe 4) band size variation was observed in the 5 isolates for both enzymes (Fig. 2B, data not shown). The results of the Southern blotting experiments confirmed that *vaa *is present as a single copy in the genome and indicated sequence variation in the *vaa *locus.

### Amplification and sequencing of the *vaa *locus of *M. hominis *132

**Figure 3:**
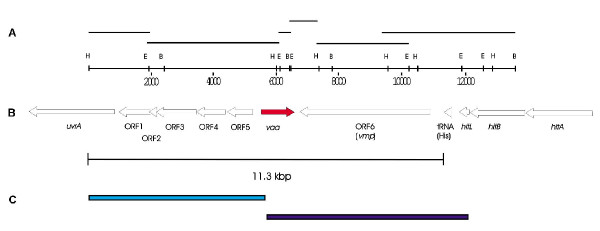
**Schematic drawing of the *vaa *locus of isolate 132. **A: Restriction map of the *vaa *locus showing the cleavage sites for *Bgl*II (B), *Eco*RI (E) and *Hin*dIII (H). Fragments amplified by inverse PCR are shown as thick lines above the restriction map. B: Open reading frames of the *vaa *locus. The *vaa *gene is shown as a red arrow and show opposite transcriptional direction compared to the other ORFs. C: The PCR products amplified from isolate 132 are shown in cyan (upstream fragment) and purple (downstream fragment).

As the mapping and restriction endonuclease analysis of *vaa *indicated a variable locus, we decided to sequence this genomic region of the *M. hominis *genome to determine the cause of the variability. A *Hin*dIII/*Eco*RI restriction map of the *vaa *locus of isolate 132 was made using the Southern blotting results (Fig. 3A). The sizes of most of the *Hin*dIII and *Eco*RI fragments including the up- and downstream regions of *vaa *were 4.2 kb or below. These fragments were thus suitable for amplification by inverse PCR. The genomic DNA was cleaved by *Hin*dIII or *Eco*RI and religated. Outward pointing oligonucleotide primers located in the *vaa *gene were used in PCR on the religated template in order to amplify regions outside the gene (Fig. 3A). PCR products of the expected sizes were observed and sequenced bidirectionally. A contig of approximately 8 kb was assembled. In order to expand the contig, a new round of inverse PCR was performed using new primer sets located in each end of the contig. The upstream region was expanded using the *Hin*dIII cleaved and religated template. To expand the downstream region, chromosomal DNA cleaved with *Bgl*II and religated was used as template. Sequencing of the expanded regions revealed that an ORF showing high similarity to the *uvrA *gene by database search was located approximately 5 kb upstream to *vaa*. The annotation of this ORF as *uvrA*, the gene encoding excinuclease ABC subunit A, was based on homology alone as this gene has not yet been characterized in mycoplasmas. Furthermore, the conserved *hitB *gene, part of the *hitABL *operon, was identified in the opposite end of the *vaa *locus (Fig. 3B). The *hitABL *operon comprises three highly conserved genes. The *hitAB *genes encode the P60 and P80 proteins, respectively. P60 and P80 form a membrane associated complex that interacts by an unknown mechanism with the evolutionary conserved, cytoplasmic HinT (histidine triad nucleotide-binding) protein encoded by *hitL*. This system was previously characterized in *M. hominis *by Henrich and coworkers [19-21]. The *hitABL *operon was located almost 5 kb downstream to the *vaa *gene of *M. hominis *132 (Fig. 3B). Thus, the *vaa *locus is bordered by highly conserved housekeeping genes. A bidirectionally sequenced contig of 11.3 kb comprising the *vaa *locus was assembled (Fig. 3C).

### Gene organization of the *vaa *locus of *M. hominins *132

Analysis of the contig harboring the *vaa *locus revealed a number of open reading frames (Fig. 3B). At the border of the sequenced upstream region the *uvrA *gene, represented by 878 bp of the 5' end of the gene, is located. Between *uvrA *and *vaa *five ORFs, numbered 1 to 5, were detected, ORF2 encoding a putative protein of only 6 kDa (Fig. 3B). This ORF was included as proteins of similar size has been detected in other bacteria [22]. The transcriptional direction of these ORFs and *uvrA *was opposite that of *vaa*. The ORFs were positioned closely, ORFs 4 and 5 had a 16 bp intergenic region, and ORFs 2 and 3 had an intergenic region of 22 bp. ORFs 3 and 4 showed an overlap of 35 bp. Thus, ORFs 1 to 5 might constitute an operon. No obvious stem-loop structures with a putative rho-independent transcriptional termination function were observed immediately downstream to any of the ORFs. The hypothetical genes were employed in database searches. The ORF adjacent to *uvrA*, (ORF1), encoding a hypothetical 35 kDa protein, showed high similarity to a range of hypothetical proteins of similar size in the database. All of these proteins contained a HAD hydrolase superfamily motif. The highest similarity was to a hypothetical protein of *Mycoplasma pulmonis *(31% identity, 54% similarity in 268 aa). No significant homologues were found for ORFs 2 to 5. ORF5 was shown to encode a hypothetical protein containing an N-terminal signal peptide with a signal peptidase II cleavage site typical of prokaryotic prolipoproteins and may thus encode a lipoprotein having a size of 29 kDa and a pI of 9.6. Interestingly, a transmembrane helix was predicted in the C-terminal part of this putative lipoprotein using the program TMHMM (membrane probability of 1 for aa 243 to 252) [23].

The downstream region of *vaa *revealed the presence of a large open reading frame of 4 kb, ORF6, encoding a hypothetical protein with a molecular weight of 160 kDa (Fig. 3B). A secretory signal peptide was identified in the N-terminal part of the putative protein using the program SignalP [24]. A signal peptidase I cleavage site was identified between A24 and S25 (mean S value of 0.956 for aa 1–27), but as no gene encoding signal peptidase I has been observed in most of the sequenced mycoplasma genomes, the protein is most likely not processed [2,17]. A homopolymeric tract of 16 thymidine residues (poly-T tract) was located 77 bp upstream to the ATG start codon of ORF6, and a putative rho-independent stem-loop terminator structure (ΔG = -16.2 kcal/mol) was observed 23 bp downstream of ORF6. The deduced aa sequence of ORF6 was used in a database search and intriguingly, high similarity to the Lmp-1 and Lmp-3 proteins of *M. hominis *was found, the highest similarity was to Lmp-1 (28% identity and 48% similarity in 1241 aa, see additional file 1). Surprisingly, homology was also found to the myosin heavy chain protein of the slime mold *Dictyostelium discoideum *(20% identity and 40% similarity in 1083 aa), and other myosin proteins in the database. In the region between ORF6 and the *hitABL *operon, a *tRNA*(His) gene was identified by database search (Fig. 3B). The highest similarity observed was to the *tRNA*(His) of *Bacillus subtilis*. Intriguingly, the 5' end of the gene displayed a high similarity to the orthologue from *Streptococcus pneumoniae *(94% identity from bp 10 to 45), even higher than to the corresponding region in *Bacillus subtilis *(92% identity) and *Mycoplasma pneumoniae *(86% identity). The transcriptional direction of ORF6, *tRNA*(His) and the *hitABL *operon was opposite that of *vaa *in analogy to the ORFs of the *vaa *upstream region (Fig. 3B).

### Variability of the *vaa *locus in 20 *M. hominis *isolates

**Figure 4:**
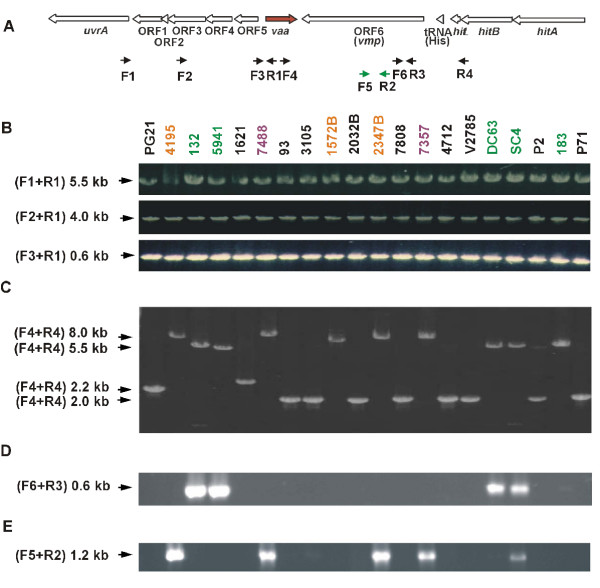
**Up- and downstream PCR analysis of 20 *M. hominis *isolates and *vmp *PCR. **A, Top: Schematic illustration of the open reading frames (indicated by open arrows) of the *vaa *locus of *M. hominis *isolate 132. Bottom: The position relative to the open reading frames of the primers used (indicated by small arrows). B, Agarose gel electrophoresis of the PCR reactions obtained using *vaa *upstream primer sets (F1, R1), (F2, R1) and (F3, R1) on the 20 *M. hominis *isolates indicated above the bands. The size of the PCR products and primer set used are indicated to the left. C, D and E, PCR reactions observed using the *vaa *downstream primer sets (F4, R4), (F6, R3) and (F5, R2), respectively. The (F6, R3) primer set was derived from *vmp-1 *and the (F5, R2) primer set (green arrows) was derived from the *vmp-2 *and *vmp-3 *genes of isolates 4195 and 7488. Isolates categorized as having *vmp-1*, *vmp-2 *and *vmp-3 *genes are indicated with green, orange and magenta coloring of letters, respectively.

To examine the variability of the *vaa *locus, PCR was performed on 20 *M. hominis *isolates representing different *vaa *types. Using a primer located in the conserved region of *vaa *and a primer located in the *uvrA *gene, a PCR product of 5.5 kb was amplified (Figs. 3 and 4B). All isolates gave rise to a band of identical size for the primer set (Fig. 4B). Furthermore, PCR was performed using the primer located in the conserved region of *vaa *and a primer located in ORF3 or ORF5. This amplified fragments of 4 kb and 0.6 kb, respectively, in all analysed isolates (Fig. 4B). The PCR results thus suggested that the upstream region is very conserved. Additionally, restriction endonuclease analysis of the 5.5 kb PCR fragment was made using the enzymes *Alu*I and *Ase*I, which cleaves at 5 and 8 sites, respectively, scattered over the entire fragment of isolate 132. This analysis revealed 13 and 3 digestion profiles, respectively, of the 20 isolates (data not shown). It was not possible to classify the profiles according to the *vaa *type or other known *M. hominis *groupings. Thus, despite a higly conserved organization and length of the ORFs in the *vaa *upstream region, there is an underlying sequence variation, presumably corresponding to the background variation level present in the *M. hominis *genome.

**Figure 5:**
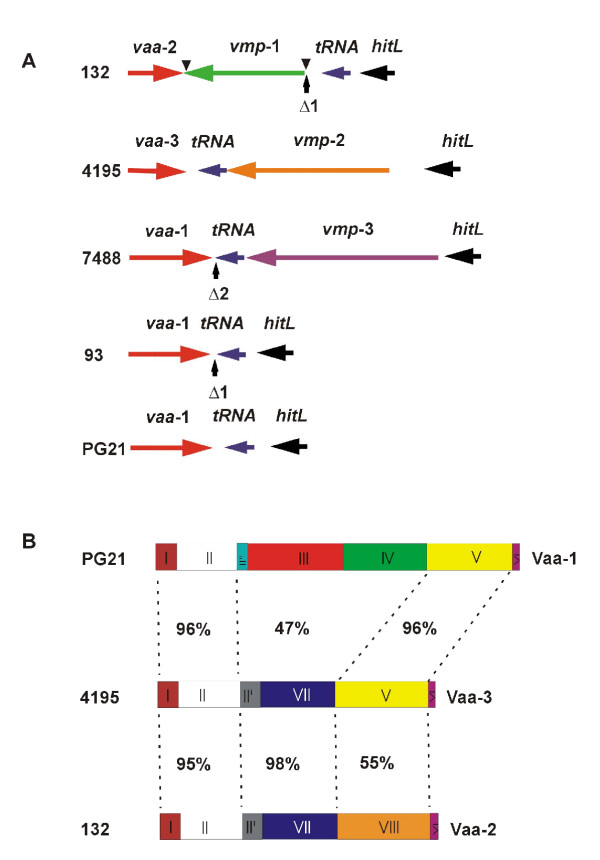
**Variation of the *vaa *downstream region. **A: Schematic drawing of the sequenced *vaa*-*hitABL *intergenic regions of the five isolates. Genetic fingerprints consisting of deletions (Δ1–2) and insertions (▼) of 90 bp upstream and 60 bp downstream to *vmp-1 *are shown. Identical *vaa *downstream regions were shared between isolates 4195 and PG21. The *vaa *gene categories are indicated by suffix -1, -3 or -2. B: Schematic comparison of the Vaa types of isolates PG21, 4195, and 132. The exchangeable cassettes are numbered III, IV, V, VII, and VIII, respectively. The percentages of identical residues between different parts of the proteins are shown.

**Figure 6:**
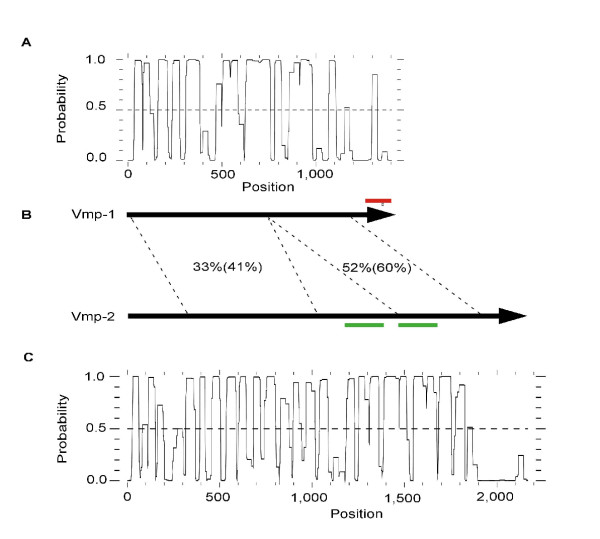
**Comparison of Vmp-1 and Vmp-2. **A and C: the predicted coiled-coil regions of Vmp-1 and Vmp-2, respectively. A threshold value of 0.5 is shown as a vertical dashed line. B: The identity (similarity) displayed by regions of the Vmp proteins. The C-terminal regions of the protein showed high mutual similarity. Green bars are shown below the repeated sequence of Vmp-2. A red bar indicates the fragment specific for Vmp-1 used for generation of a polyclonal antibody.

In contrast, when primers located in the conserved region of *vaa *and *hitB*, respectively, were used in PCR, a pattern revealing different product sizes was observed (Fig. 4C). The expected 5.5 kb PCR product was amplified in 5 isolates, 10 gave rise to bands of approximately 2 kb and 5 gave rise to larger bands of 7.5 and 8 kb (Fig. 4C). The size of the 2 kb products corresponded to the deletion of ORF6. This was verified by sequencing of the products from *M. hominis *PG21 and 93 (Fig. 5). Sequencing of the 8 kb and 7.5 kb PCR products from *M. hominis *7488 and 4195, respectively, revealed ORFs of 6.5 kb and 5.5 kb showing homology to ORF6 (Figs. 5 and 6). Because of the apparent variability of this protein, ORF6 and the corresponding ORFs of *M. hominis *7488 and 4195 were named *vmp *(variable membrane protein). The isolates were divided into *vmp *groups based on the above PCR results according to the size of the *vmp *gene observed in the different sized downstream PCR products. The *vmp *gene having a size of 4 kb was named *vmp *category 1 or simply *vmp*-1, and the isolates (indicated with green numbers in Fig. 4) giving rise to a 5.5 kb downstream PCR product, containing the *vmp-1 *gene, were categorized as having a *vmp-1 *gene type (Fig. 4C). Likewise, the *vmp *genes having a size of 5.5 kb and 6.5 kb were named *vmp*-2 and *vmp*-3, respectively, and the isolates giving rise to downstream PCR products of 7.5 and 8 kb (indicated in Fig. 4 by orange and magenta numbers, respectively) were categorized as having a *vmp-2 *and *vmp-3 *gene type, respectively (Fig. 4C). It was not possible to design a universal *vmp *primer set that would amplify a fragment from all the sequenced *vmp *genes due to the high sequence variation observed between the genes. Instead, a primer set amplifying a 0.6 kb fragment of *vmp-1 *was used for PCR of the 20 isolates. Four of the five genes categorized as *vmp-1 *gave rise to a strong band of 0.6 kb (Fig. 4D). The isolate 183 also categorized as having a *vmp-1 *gene type gave rise to a very faint band of 0.6 kb. A second primer set was designed that would amplify a 1.2 kb fragment from both *vmp-2 *and *vmp-3 *(Fig. 4E). Again, only four of the five isolates expected gave a product. The isolate SC4 categorized as having a *vmp-1 *gene type also gave a faint band with the *vmp-2/3 *primer set. The isolate 1572B did not give a product with the primer set, but was categorized as having a *vmp-2 *gene type because it gave rise to a downstream PCR product of approximately 7.5 kb. Thorough examination revealed that the size of the 1572B downstream PCR product was slightly smaller than that of isolate 4195. Thus, isolate 1572B may harbor a fourth *vmp *type. The *vmp-2 *and *vmp-3 *genes were flanked by a poly-T tract and a stem-loop terminator structure (ΔGs of -16.7 and -15.6 kcal/mol, respectively) showing high homology of the stem regions to that observed for *vmp-1*. The stem-loop structure was located approximately 500 bp downstream of the stop codon of *vmp-2 *but interestingly, the homology between *vmp-*2 and *vmp*-3 extends beyond the stop codon in *vmp*-2. Careful analysis reveals that a poly-A tract in the 3'-end of the *vmp *genes has an extra (9 total) adenine in *vmp*-2 compared to *vmp*-3, which causes a premature termination of translation of the *vmp*-2 gene. If the extra adenine of the poly-A tract was deleted the ORF would continue for approximately 500 bp, corresponding to *vmp*-3, and the termination codon would be situated close the putative rho-independent transcriptional termination stem-loop structure. The remaining 10 isolates were divided into two groups based on a genetic fingerprint in the *vaa*-*hitABL *intergenic region of isolates lacking *vmp *(Fig. 5). The genetic fingerprint was an insertion/deletion in the intergenic regions downstream to *vaa*. The *vaa-hitABL *intergenic region of isolate 93 was nearly identical to the corresponding region of isolate PG21, except for a deletion of approximately 300 bp shortly after the *vaa *terminator stem-loop structure (Δ1, Fig. 5). Two isolates (PG21 and 1621) gave the 2.2 kb downstream PCR product, whereas the remaining 8 isolates gave the 2 kb downstream PCR product of isolate 93 (Fig. 4B). The *vmp *type or absence of the *vmp *gene showed no correlation to the *vaa *type of the isolates. Surprisingly, the position of the *vmp *gene relative to the *tRNA*(His) gene was different for *vmp*-1 and *vmp-2*, the latter positioned between *tRNA*(His) and *hitABL*, whereas the more similar *vmp*-2 and *vmp*-3 genes were positioned identically (Fig. 5). A careful sequence analysis was performed on the insertion/deletion sites of the *vmp *genes to try to deduce a mechanism of insertion/deletion. The *vmp-2 *and *vmp-3 *genes seemed to be inserted at the poly-T tract in the 5' end and stem-loop terminator structure at the 3' end. When compared to the *vaa-hitABL *intergenic region of isolate PG21, the sequences flanking the insertion sites were identical in isolates 7488/4195 and PG21 and insertion did not result in deletion of part of the intergenic sequence. The insertion site was A/T rich, and sequence identity was observed in a small 8 bp thymidine-rich (*vmp *coding strand) sequence box between the insertion site in isolate PG21 and the stem region of the stem-loops of *vmp-2 *and *vmp-3*. Furthermore, a 165 bp deletion (Δ2 in Fig. 5) was observed in the *vaa-tRNA*(His) intergenic region of isolate 7488 compared to isolate PG21. This deletion was located immediately downstream to the region deleted in isolate 93. In contrast, analysis of the *vmp-1 *insertion site did not show insertion at the stem-loop and poly-T structures when compared to the *vaa-hitABL *intergenic regions of isolates PG21 and 93. Sequence regions of 60 bp downstream to the stem-loop structure and 90 bp upstream to the poly-T tract which did not show any homology to the *vaa-hitABL *intergenic region of isolate PG21 were observed at the borders of the *vmp-1 *gene. Interestingly, comparison of the insertion site of the *vmp-1 *region including the 60 bp and 90 bp bordering sequences with the *vaa-hitABL *intergenic region of isolate PG21 revealed that insertion resulted in a deletion in the intergenic sequence corresponding to the 300 bp deletion (Δ1 in Fig. 5) observed for isolate 93. The sequences flanking this region in isolate PG21 was A/T rich but did not show high homology to each other or to the insertion site of the *vmp-2 *and *vmp-3 *genes. In conclusion, no obvious direct or inverted repeat structures were observed indicating a mechanism of insertion other than homologous recombination. Isolates PG21 and 4195 shared identical *vaa-hitABL *intergenic regions outside the *vmp-2 *insertion (Fig. 5). These isolates carry distinct *vaa *gene types, PG21 having a three cassette *vaa*-1 type and 4195 having a two cassette *vaa*-3 type [13]. The distal 3' end cassette of these *vaa *genes shows high mutual similarity, whereas the remaining exchangeable cassettes of both genes are distinct (Fig. 5B). The 5' end including the first cassette of the *vaa *gene of isolate 4195 shows high similarity to the corresponding part of the *vaa *gene of isolate 132, harboring a different two cassette *vaa *gene type. The 3' end distal cassette of *vaa *from isolate 132 differs from that of isolates PG21 and 4195. Thus, the *vaa *type of isolate 4195 seems to be a chimera of the *vaa *types of isolates 132 and PG21 (Fig. 5B).

The GC content of the *vaa *and *vmp *genes of isolate 132 were 27% and 25% compared to a total of 25% for the 11.3 kb contig.

### Characterization and detection of the Vmp protein

Alignments of the deduced Vmp-1 and Vmp-3 protein sequences revealed highest similarity in the C-terminal part (52% identity and 60% similarity in 382 aa). Half of this region was repeated once in Vmp-3 (Fig. 6B). The N-terminal part showed a lower, but significant overall similarity (33% identity and 41% similarity in 660 aa). In contrast, Vmp-2 and Vmp-3 showed almost complete conservation of the N-terminal part (93% identity in 1552 aa), whereas the C-terminal part showed a similarity corresponding to that between the Vmp-1 and Vmp-3 (55% identity and 69% similarity in 276 aa). The repeated region of Vmp-3 was only present in one copy in Vmp-2.

Sequence analysis revealed that the deduced Vmp proteins have a predominantly alpha-helical structure, and a coiled-coil region extending throughout almost the entire length of the proteins was identified (Fig. 6A and 6C). In Vmp-1 the coiled-coil motif extended from residues 35 to 1328 out of a total of 1404 residues. Likewise, the coiled-coil region of Vmp-2 spanned residues 28 to 1631 out of 1829 and residues 29 to 1869 out of 2168 residues of Vmp-3. The coiled-coil region contained numerous short disruptions of the predicted coiled-coil and alpha-helical structure (Fig. 6A and 6C). As this region showed high similarity to the Lmp proteins, the Lmp sequences were analysed for the presence of a coiled-coil region. Intriguingly, the Lmp proteins were shown to contain a coiled-coil region extending throughout most of the protein in analogy to the Vmps (data not shown).

**Figure 7:**
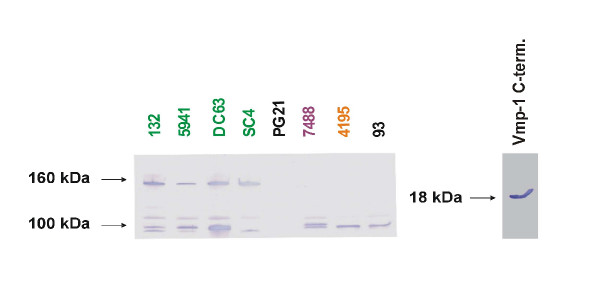
**Detection of Vmp-1. **Immunoblotting of selected isolates using the polyclonal antisera generated against the C-terminal part of Vmp-1 from isolate 132. Isolates 132, 5941, DC63 and SC4 show a sharp band around 160 kDa, thus demonstrating the expression of Vmp-1 in these isolates. The polyclonal antiserum additionally reacted with multiple bands around 100 kDa. These bands probably corresponds to a ~100 kDa *M. hominis *protein which previously has been shown to bind Ig-molecules unspecifically. This protein has not been observed in PG21. The right panel shows the coomassie stained, recombinant Vmp-1 C-terminal fragment used for immunization.

A polyclonal antibody was raised against the distal C-terminal part of Vmp-1 from isolate 132 (Fig. 6B). This region shows low homology to the Vmp-2 and Vmp-3, and the antibody was applied in immunoblotting using antigen from 8 selected isolates (Fig. 7). The antibody reacted with a protein of 160 kDa from isolates 132, 5941, DC63 and SC4, in agreement with the predicted molecular weight of the protein encoded by the *vmp*-1 gene of these isolates. These data thus demonstrate that Vmp is expressed in *M. hominis*. Furthermore, the antibody reacted with a 100 kDa protein in all isolates except PG21. This is presumably a protein that has been shown previously to bind Ig-molecules unspecifically, and is not present in PG21 [25]. Apart from the 100 kDa protein the isolates PG21 and 93, having no *vmp *gene, and isolates 4195 and 7488 having a Vmp-2 and Vmp-3 type, respectively, did not react with the antibody, as expected from the low homology of the C-terminal region (Fig. 7).

## Discussion

The presented analysis of the *vaa *locus reveals that it is a highly dynamic region of the *M. hominis *genome. The data indicate that variation in the 3' end of the *vaa *gene may be attributed recombinational events involving regions outside the gene. The insertion/deletion of the *vmp *gene downstream to *vaa *in different positions relative to the *tRNA*(His) gene, and the lack of correlation of *vmp *type and absence of *vmp *to *vaa *type suggests frequent recombination in this locus (Fig. 5). The finding that the two distinct *vaa *types of isolates PG21 and 4195 share 3' end cassettes and homologous downstream intergenic sequences, suggests that the *vaa *type of 4195 arose from intraspecies recombination (Fig. 5B). This event might have taken place between a *M. hominis *cell carrying a *vaa *type with a 5'end analogous to the *vaa *type of isolate 132 and a cell harboring a *vaa *type with a 3' end similar to that of PG21. Another *M. hominis *gene encoding a surface exposed lipoprotein, P120, was shown to contain a hypervariable and two semivariable domains [26]. This gene maps in close proximity to the *vaa *locus in the *M. hominis *genome (Fig. 1A). The groups of isolates carrying a specific hypervariable domain did not correlate with the *vaa *type [13,26]. Thus it is plausible that this region of the *M. hominis *genome harboring most of the characterized genes for membrane proteins is a genomic plasticity zone as previously suggested [18].

Based on these findings, recombination between different *M. hominis *subpopulations may be a general mechanism for the generation of antigen variation in this mycoplasma species. A necessity for intraspecies recombination is the transfer of DNA. No plasmids or phages have been observed for *M. hominis*, but the transfer of Tn*916*, presumably by conjugation, from a *Streptococcus faecalis *donor has been shown with low efficiency [27,28]. Conjugation between *M. hominis *cells has not been demonstrated. Chemically competent *M. hominis *were shown to be capable of uptake of homologous naked DNA. This was demonstrated by the transfer of tetracycline resistance from the DNA of a tetracycline resistant *M. hominis *isolate to a competent, sensitive isolate [29]. Thus, the knowledge regarding mechanisms of DNA uptake in *M. hominis *is sparse, and this subject needs to be addressed.

The location of *vmp *in close proximity to a *tRNA *gene is analogous to genetic elements of other bacteria. These elements, often referred to as genetic islets (<10 kb) or genetic islands (>10 kb), are variable sites when comparing genomes of different isolates of a given species. Often, genetic islets/islands carry pathogenesis factors and are specific for virulent clones of the species. Such factors include adhesins, toxins and restriction/modification systems. Frequently, the genetic elements are inserted into *tRNA *genes, show a GC content diverging from the surrounding regions and are flanked by repeated elements [30]. Although the *vmp *gene was located on either side of the *tRNA*(His) gene in isolates 132 and 7488, respectively, the GC content of *vmp *was similar to the remaining part of the *vaa *locus analyzed and the *M. hominis *genome in general (28%) and despite a thorough analysis, no obvious flanking structures such as direct or inverted repeats were observed which could indicate a site specific mechanism of insertion. Thus, the *vmp *gene may be mycoplasma specific and insertion/deletion of *vmp *at the *vaa *locus seems mediated by homologous recombination.

It was possible to amplify *vmp *fragments by PCR from four out of five isolates categorized as having a *vmp-1 *gene type and likewise for four out of five isolates having a *vmp-2 *or *vmp-3 *gene type. The pronounced heterogeneity observed between the *vmp *genes could explain the missing reaction of the remaining two isolates as being caused by sequence variation of the individual gene types as observed for a number of other *M. hominis *membrane protein genes, but it is also possible that additional *vmp *gene types exists.

The size and predicted structure of Vmp is interesting. The coiled-coil motif extending through most of the protein, is a highly versatile motif involved in protein-protein interactions [31]. This motif is found in proteins carrying out diverse functions such as structural proteins, transcription factors, translation factors and extracellular proteins [32]. The coiled-coil domains often mediate the oligomerization of proteins forming di-, tri-, tetra- or even pentamers [31]. Additionally, coiled-coil motifs in some cases are involved in intramolecular interactions forming highly stable, ridgid and compact structures. The length of the coiled-coil region in the primary structure of Vmp is comparable to that of eukaryotic class II myosins, proteins involved in movement along actin filaments [33]. Furthermore, a coiled-coil region of similar length was observed for the cytadherence related HMW2 protein of *Mycoplasma pneumoniae*. This protein is cytoplasmic, part of the primitive cytoskeleton of this mycoplasma and truncation of the gene resulted in loss of cytadherence [34]. HMW2 is believed to form dimers due to the coiled-coil region [35]. In contrast to HMW2, Vmp contains a C-terminal region having no coiled-coil motifs. Furthermore, a signal sequence involved in transmembrane translocation of proteins was identified in the Vmps, but not in HMW2. Thus, it is highly likely that the Vmp protein is located on the cell surface of *M. hominis *in contrast to the cytoplasmic HMW2.

The identification of a novel putative membrane protein displaying sequence variation is intriguing, and furthermore the remarkable size and structure displayed by the Vmp protein is interesting and should prompt investigations on the biological function of this protein in *M. hominis*.

## Conclusions

We have demonstrated that in some isolates, the *vaa *locus of *M. hominis *contains a divergent genetic islet encoding a large, putative membrane protein called Variable membrane protein (Vmp). This genetic islet is only present in the locus of half of the 20 islolates tested, and three distinct, homologous Vmp types were observed. The composition of the locus was analysed and it was found that the *vaa *gene has a conserved upstream region and a highly variable downstream region, which contains the genetic islet. This locus organization corresponds to the organization of the *vaa *gene itself having a conserved 5' end and a variable 3' end. Thus, the mechanism underlying variation of the *vaa *gene seems to be intraspecies recombination exchanging variable regions of *vaa *and downstream regions of *vaa*, giving rise to a variable and dynamic 'hot spot' in the *M. hominis *genome.

## Methods

### Isolates and growth media

Twenty *M. hominis *isolates were analysed (PG21, 4195, 132, 5941, 1621, 7488, 93, 3105, 1572B, 2032B, 2347B, 7808, 7357, 4712, V2785, DC63, SC4, P2, 183, and P71). The *M. hominis *isolates were cultivated in BEa medium (heart infusion broth (Difco), 2.2% (w/v); horse serum, 15% (v/v); fresh yeast extract, 1.9% (w/v); benzylpenicillin, 40 IU ml^-1^; L-arginine, 0.23% (w/v); phenol red 0.0023% (w/v)). The pH of the medium was adjusted to 7.2 and the medium was sterilized by filtration [36]. The *M. hominis *isolates were harvested by centrifugation at 15,000 rpm for 45 min at culture volumes greater than 1.5 ml or at 20,000 rpm for 15 min for culture volumes smaller than 1.5 ml. *E. coli *OneShot competent cells and the pCRII plasmid vectors were used for TA-cloning (Invitrogen).

### Pulsed field gel electrophoresis (PFGE)

PFGE was performed on genomic DNA from the five *M. hominis *isolates PG21, 4195, 132, 93 and 7488. The *M. hominis *isolates were grown in BEa medium to log phase and harvested. The cell pellets were washed and resuspended in PBS buffer (20 mM sodium phosphate, 250 mM NaCl, pH 7.4). Melted NA agarose (Amersham Pharmacia Biotech) was mixed with the cell suspension in a plastic mold on ice. The agaroseblocks hereby formed were incubated overnight with 1 mg/ml Proteinase K (Roche) in lysis buffer (1% Sarcosyl, 0.5 M EDTA, 10 mM Tris HCl, pH 9.5). Subsequently, each block was washed twice in lysis buffer followed by one wash in TE buffer (10 mM Tris HCl, 1 mM EDTA, pH 8.0) and cut into eight blocks of identical size. The blocks were digested overnight with 40 units of one of five restriction enzymes (*Sma*I, *Bam*HI, *Xho*I, *Sal*I, and *Apa*I) and 12 mg of BSA. Subsequently, the blocks were inserted into the slots of 1% NA agarose gels and the holes sealed with melted NA agarose. *Hin*dIII digested λ DNA and a λ DNA ladder (FMC) was used as molecular weight markers. The gels were used for PFGE using the CHEF-DRII separation system (Bio-Rad).

### Preparation of DNA

DNA from the *M. hominis *isolates was isolated using the method described in [37]. Briefly, *M. hominis *cells were harvested and subsequently lysed on ice in a buffer containing 0.7% (w/v) N-laurylsarcocine, 10 mg RNase ml^-1 ^(Sigma), 20 mM Tris pH 7.5 and 20 mM EDTA. Proteinase K (150 mg ml^-1^) was added and the cell lysate was incubated at 55°C for 2 hrs and 37°C for 1–2 hrs followed by phenol, phenol/chloroform and chloroform extractions [38]. DNA preparations from the isolates 1572B, 2032B and 2347B were made by Proteinase K (150 mg ml^-1^) treatment of harvested *M. hominis *at 55°C for 1 h. After the incubation the solution was heated to 100°C for 5 min to inactivate the enzyme. Plasmids from transformed *E. coli *were prepared as described in [38], for sequencing the phenol/chloroform extraction was omitted.

### Southern blotting

PCR-products derived from different *vaa *types with sizes of 1220 bp (probe 1), 600 bp (probe 2), 800 bp (probe 3), and 660 bp (probe 4) were used for TA-cloning, performed according to manufacturers instructions (Invitrogen) [13]. Probe 1 contained most of the *vaa-1 *gene from *M. hominis *7808, including the three cassettes III, IV and V [13]. Probe 2 contained the conserved 5' end and cassette III from the *vaa-1 *gene of *M. hominis *PG21 and probe 3 contained most of the *vaa-3 *gene of *M. hominis *V2785 including cassettes V and VII. TA-cloned PCR fragments or linear PCR fragments were used as DNA probes and labeled with radioactive (α-^32^P)dATP by nick-translation, performed as follows. 0.5–1 μg DNA was mixed with 1 × nick-translation buffer (50 mM Tris HCL (pH 7.2), 10 mM MgSO_4_, 0.1 mM DTT, 50 mg BSA, 60 mM of dTTP, dCTP, and dGTP respectively, 5 units of DNA polymerase I (Gibco), 0.5 ng DNase I (Roche), 20 mCi (α-^32^P)dATP (Du Pont) and ddH_2_O up to 50 μl. The reaction was incubated at 14–16°C for 1 h. Incorporation of radioactive nucleotides was verified by TLC and terminated by addition of TE-buffer with 0.5 M EDTA. The radioactive probes were denatured by heating to 100°C for 5 min and hybridization performed in 2 × SSC (1 × SSC is 0.15 M NaCl and 0.015 M sodium citrate), 0,5% SDS, 100 mg/ml yeast RNA, 5 × Denhardts solution (0.1% Ficoll, 0.1% BSA, 0.1% polyvinyl pyrollidone (Serva)) at 60°C in a hybridization oven. The membranes were washed in 6 × SSC and 0.5% SDS. The membranes were placed in sealed plastic bags and exposures of X-ray films were performed at room temperature or at -20°C.

Genomic DNA samples of the isolates PG21, 4195, 132, 93 and 7488 were cleaved with either *Hin*dIII or *Eco*RI and separated on 0.7% agarose gels. The gels were stained with ethidium bromide and photographed under UV irradiation. Preceding the alkaline denaturation, partial hydrolysis of the DNA in the PFGE gels was performed by soaking in a 0.25 M HCl solution to enhance the transfer of large DNA fragments. DNA transfer to Hybond-N membranes (Amersham Biosciences) was carried out as described in [38].

### PCR

PCR was performed using the Expand™ High Fidelity PCR System from Roche according to the manufacturer's instructions, except for the amplification of the 1.2 kb *vmp-2/3 *PCR product where Taq polymerase was used (PE Biosystems). Custom oligonucleotide primers were purchased from DNA Technology (Aarhus, Denmark). PCR products were purified using the Wizard kit (Promega) according to manufacturers instructions. PCR conditions used for inverse PCR and amplification of downstream and upstream regions of the 20 isolates were as follows: 2 min at 92°C, 10 cycles of 10 s at 92°C, 30 s at 55°C, 8 min at 68°C, 20 cycles of 10 s at 92°C, 30 s at 55°C, 8 min at 68°C with 5 s added to the elongation time pr. cycle. Finally, an extension step of 7 min at 68°C was performed. The primers used for amplification of the 5.5 kb upstream product were F1 (CAGTACATGTTAATCCCAGAA GTATAGTTGG) and R1 (GCTGGATAATCGCCGTATGAACCTGC). The R1 primer was also used for amplification of the 4 kb and 0.6 kb PCR products in combination with the primers F2 (GGATCTTCTTTGTGGTCTTCC) and F3 (GGGATAGTTAGTAAAG TTGGAATAGCC), respectively. For amplification of the downstream region in the 20 isolates, the primers F4 (GCAGGTTCATACGGCGATTATCCAGC) and R4 (GCCACTTGCGGTTCTTCC) were used. For the amplification of the 0.6 kb *vmp-1 *PCR product, the primers F6 (CCACTGATACGTGATTTAAAAAGAAAAG) and R3 (GGTATTGTTTCTTTATCTAAGATGTTTTCAAATTC) were used with the following PCR conditions: 4 min at 94°C, 30 cycles of 15 s at 94°C, 30 s at 50°C, 1 min at 72°C and a final extension of 5 min at 72°C. For amplification of the 1.2 kb *vmp-2/3 *PCR product, similar conditions were used with an annealing temperature of 57°C and an elongation time of 2 min, and the primers were F5 (GAACAATTAAAAACATTAATTGGCTTAA GTGATG) and R2 (GTTTTATCTACATTGTTTTCGGATAAGG).

### Restriction endonuclease analysis

The 5.5 kb upstream PCR products from the 20 analysed isolates were subjected to restriction endonuclease analysis employing the enzymes *Alu*I and *Ase*I (New England Biolabs) according to manufacturers instructions and analysed on 1 × TBE/2% agarose gels.

### Sequencing

Sequencing reactions were carried out bidirectionally using the ABI PRISM Dye Terminator Cycle Sequencing Ready Reaction Kit (Perkin Elmer) on purified plasmid DNA (TA-cloned PCR products) or directly on the purified PCR products according to the instructions supplied by the manufacturer. Sequencing was performed on an ABI PRISM 377 DNA Sequencer from Perkin Elmer.

### Cloning, expression and generation of polyclonal antibodies to a Vmp-1 fragment

Oligonucleotide primers (DNA technology, Aarhus, Denmark) were designed in order to amplify by PCR, the region of *vmp*-1 from isolate 132 encoding aa 1281 to 1404 of the Vmp-1 protein. Cloning and expression of the construct was performed using the pET-30 Ek/LIC vector according to manufacturers instructions (Novagen, Madison, USA). The His-tagged fusion protein was purified using a nickel chelated column (High Trap Sepharose, Amersham Pharmacia Biotech) under denaturing conditions as previously described [39]. Sera containing polyclonal antibody directed against the C-terminal Vmp-1 fragment was obtained by immunizing a rabbit three times intramuscularly with 20 mg of recombinant protein dissolved in Freunds complete adjuvant and three times intravenously with 20 mg protein dissolved in PBS. SDS-PAGE and immunoblotting were performed as previously described [13].

### Computer analysis

Computer analysis of the obtained DNA sequences was performed using the Wisconsin Package Version 9.0, Genetics Computer Group (GCG), Madison, Wisc., sequence analysis software package [40]. Data base searches were performed using both NetBlast and FastA. Furthermore, the programs SignalP and TMHMM, both found at the website , were used to predict signal sequences and transmembrane helices, respectively [23,24]. Energy of putative rho-independent stem-loop terminator structures was calculated using the RNA mfold server at [41].

### Accession numbers

The DNA sequences obtained in this study were deposited to the EMBL database under the following accession numbers: AJ416752, AJ545046 AJ629113, AJ629114 and AJ629115.

## Authors' contributions

The individual parts of the work presented in the paper were conducted as follows: The ideas and designs of the experiments were developed by all the authors. Pulsed field gel electrophoresis and Southern blottings were performed by JE and TB. PCR, sequencing, and sequence analysis were performed by TB and AB. The fusion protein, polyclonal antisera and immunoblotting were made by TB. The manuscript was primarily written by TB and discussed with and approved by all authors.

## Supplementary Material

Additional File 1 Multiple sequence alignment. Alignment of the three Vmp types and Lmp1 and Lmp3 from type strain PG21 using ClustalW. Click here for file

## References

[B1] Clyde Jr WA, Chanock RM, Tully. JG, Davis B D, R Dulbecco, H N Eisen and H S Ginsberg (1990). Mycoplasmas. Microbiology.

[B2] Razin S, Yogev D, Naot Y (1998). Molecular biology and pathogenicity of mycoplasmas. Microbiol Mol Biol Rev.

[B3] Ladefoged SA (2000). Molecular dissection of Mycoplasma hominis. APMIS Suppl.

[B4] Christiansen G, Maniloff J, R N McElhaney, L R Finch and J B Baseman (1992). Genetic variation in natural populations. Mycoplasmas: molecular biology and pathogenesis.

[B5] Christiansen G, Ladefoged S, Hauge S, Birkelund S, Andersen. H, Stanek G GH Cassell JG Tully and RF Whitcomb (1990). Use of monoclonal antibodies for detection of antigen variation in Mycoplasma hominis.. Recent advances in mycoplasmology.

[B6] Andersen H, Birkelund S, Christiansen G, Freundt EA (1987). Electrophoretic analysis of proteins from Mycoplasma hominis strains detected by SDS-PAGE, two-dimensional gel electrophoresis and immunoblotting. J Gen Microbiol.

[B7] Olson LD, Renshaw CA, Shane SW, Barile MF (1991). Successive synovial Mycoplasma hominis isolates exhibit apparent antigenic variation. Infect Immun.

[B8] Ladefoged SA, Christiansen G (1992). Physical and genetic mapping of the genomes of five Mycoplasma hominis strains by pulsed-field gel electrophoresis. J Bacteriol.

[B9] Sogaard IZ (2001). Recombination in Mycoplasma hominis. Department of Medical Microbiology and Immunology.

[B10] Jensen LT, Ladefoged S, Birkelund S, Christiansen G (1995). Selection of Mycoplasma hominis PG21 deletion mutants by cultivation in the presence of monoclonal antibody 552. Infect Immun.

[B11] Zhang Q, Wise KS (1996). Molecular basis of size and antigenic variation of a Mycoplasma hominis adhesin encoded by divergent vaa genes. Infect Immun.

[B12] Zhang Q, Wise KS (1997). Localized reversible frameshift mutation in an adhesin gene confers a phase-variable adherence phenotype in mycoplasma. Mol Microbiol.

[B13] Boesen T, Emmersen J, Jensen LT, Ladefoged SA, Thorsen P, Birkelund S, Christiansen G (1998). The Mycoplasma hominis vaa gene displays a mosaic gene structure. Mol Microbiol.

[B14] Henrich B, Lang K, Kitzerow A, MacKenzie C, Hadding U (1998). Truncation as a novel form of variation of the p50 gene in Mycoplasma hominis. Microbiology.

[B15] Henrich B, Kitzerow A, Feldmann RC, Schaal H, Hadding U (1996). Repetitive elements of the Mycoplasma hominis adhesin p50 can be differentiated by monoclonal antibodies. Infect Immun.

[B16] Boesen T, Fedosova NU, Kjeldgaard M, Birkelund S, Christiansen G (2001). Molecular design of Mycoplasma hominis Vaa adhesin. Protein Sci.

[B17] Ladefoged SA, Christiansen G (1998). Mycoplasma hominis expresses two variants of a cell-surface protein, one a lipoprotein, and one not. Microbiology.

[B18] Mygind T, Birkelund S, Christiansen G (2000). Characterization of the variability of a 75-kDa membrane protein in Mycoplasma hominis. FEMS Microbiol Lett.

[B19] Kitzerow A, Henrich B (2001). The cytosolic HinT protein of Mycoplasma hominis interacts with two membrane proteins. Mol Microbiol.

[B20] Henrich B, Berns G, Weinhold M, Kitzerow A, Schaal H, Hadding U (1998). Cloning and expression of P60, a conserved surface-localized protein of Mycoplasma hominis, in Escherichia coli. Biol Chem.

[B21] Henrich B, Feldmann RC, Hadding U (1993). Cytoadhesins of Mycoplasma hominis. Infect Immun.

[B22] Shaw AC, Larsen MR, Roepstorff P, Christiansen G, Birkelund S (2002). Identification and characterization of a novel Chlamydia trachomatis reticulate body protein. FEMS Microbiol Lett.

[B23] Krogh A, Larsson B, von Heijne G, Sonnhammer EL (2001). Predicting transmembrane protein topology with a hidden Markov model: application to complete genomes. J Mol Biol.

[B24] Nielsen H, Engelbrecht J, Brunak S, von Heijne G (1997). Identification of prokaryotic and eukaryotic signal peptides and prediction of their cleavage sites. Protein Eng.

[B25] Moller SA, Birkelund S, Borrebaeck CA (1990). A high-affinity human monoclonal IgM antibody reacting with multiple strains of Mycoplasma hominis. J Clin Lab Anal.

[B26] Nyvold C, Birkelund S, Christiansen G (1997). The Mycoplasma hominis P120 membrane protein contains a 216 amino acid hypervariable domain that is recognized by the human humoral immune response. Microbiology.

[B27] Roberts MC, Kenny GE (1987). Conjugal transfer of transposon Tn916 from Streptococcus faecalis to Mycoplasma hominis. J Bacteriol.

[B28] Saha A, Cerone AM, Furness G (1982). Attempts to detect by physicochemical methods plasmid DNA in mycoplasmas of human origin before and after transformation to tetracycline resistance. Can J Microbiol.

[B29] Furness G, Cerone AM (1979). Preparation of competent single-cell suspensions of Mycoplasma hominis tets and Mycoplasma salivarium tets for genetic transformation to tetracycline resistance by DNA extracted from Mycoplamsa hominis tetr. J Infect Dis.

[B30] Hacker J, Carniel E (2001). Ecological fitness, genomic islands and bacterial pathogenicity. A Darwinian view of the evolution of microbes. EMBO Rep.

[B31] Lupas A (1996). Coiled coils: new structures and new functions. Trends Biochem Sci.

[B32] Burkhard P, Stetefeld J, Strelkov SV (2001). Coiled coils: a highly versatile protein folding motif. Trends Cell Biol.

[B33] Sellers JR (2000). Myosins: a diverse superfamily. Biochim Biophys Acta.

[B34] Reddy SP, Rasmussen WG, Baseman JB (1996). Isolation and characterization of transposon Tn4001-generated, cytadherence-deficient transformants of Mycoplasma pneumoniae and Mycoplasma genitalium. FEMS Immunol Med Microbiol.

[B35] Krause DC, Proft T, Hedreyda CT, Hilbert H, Plagens H, Herrmann R (1997). Transposon mutagenesis reinforces the correlation between Mycoplasma pneumoniae cytoskeletal protein HMW2 and cytadherence. J Bacteriol.

[B36] Christiansen G, Andersen H (1988). Heterogeneity among Mycoplasma hominis isolates as detected by probes containing parts of ribosomal ribonucleic acid genes. Int J Syst Bacteriol.

[B37] McClenaghan M, Herring AJ, Aitken ID (1984). Comparison of Chlamydia psittaci isolates by DNA restriction endonuclease analysis. Infect Immun.

[B38] Sambrook J, Fritsch EF, Maniatis. T (1989). Molecular cloning: a laboratory manual.

[B39] Mygind P, Christiansen G, Persson K, Birkelund S (1998). Analysis of the humoral immune response to Chlamydia outer membrane protein 2. Clin Diagn Lab Immunol.

[B40] Devereux J, Haeberli P, Smithies O (1984). A comprehensive set of sequence analysis programs for the VAX. Nucleic Acids Res.

[B41] Zuker M (2003). Mfold web server for nucleic acid folding and hybridization prediction. Nucleic Acids Res.

