# Supplementary material for: The vaa locus of Mycoplasma hominis contains a divergent genetic islet encoding a putative membrane protein
Source: BMC Microbiol. 2004 Sep 22;4:37. doi: 10.1186/1471-2180-4-37 (PMC524362; doi:10.1186/1471-2180-4-37)
Supplement: Additional File 1 — Multiple sequence alignment. Alignment of the three Vmp types and Lmp1 and Lmp3 from type strain PG21 using ClustalW. [file 1471-2180-4-37-S1.pdf]

```
                     10         20         30         40         50         60
  1    MNKKIGILLSISLLATAATTATIAAIAAKGKFKKQQELKQNIEKSKAELNKAKSILEKL   Vmp-2
  1    MNKKIGILLSISLLATAATTATIAAIAAKGKFKKQQELKQNIEKSKAELNKAKSILEKL   Vmp-3
  1    MKKKLNILLSISLIAAAASTAAIASVCASKNNKFRKQRKYNKETEKQEINQAKENLKKL   Vmp-1
  1   MNEKKKKIAIPLAILCGGLAIATTALIAIKAR.RHKIANQSQKENLLQNFKKLQKQLNEL   Lmp-1
  1    MKKRINILMSISIVCAAASTSAIAAMCSISN.NSTKKYNEAKSRLLNLIKKLDNE....   Lmp-3

                     70         80         90         100        110        120
 60   LNSDYAKNVNKLEESKILD.NAKIDENSSLQDIKNKTKQVKEALNSLTQKILERKQKEQQ   Vmp-2
 60   LKSDYAKNVNKLEESKILD.NAKLNENSSLQEIKNKTKQVKEAINSLTQKILERKQNEQQ   Vmp-3
 60   LELIKSSDIDKTNESKVLD.NSKIDENSSIEDIQNKTQEIEKAIESLTKKINDKNKKHEE   Vmp-1
 60   LGYKIVNEINVFHEQEVLQGSLKINNKSETKAIEEETLRLKDAITLLISKIKNQINQKE.   Lmp-1
 55   .GQKKANDFIARQDKKFNSTAFKNHSN.TSKLIDEIEIFSKKILENLQKDEK....QRL.   Lmp-3

                     130        140        150        160        170        180
119   NQNDKKTLDKLRLDFANAQEELKALINSKDGKSVDTTKAVEVLKNTKITNSSATFQVLPA   Vmp-2
119   NQNDKKTLDKLRLDFANVKEELQALINSKDGKSVDTTKATEVLKNTKITNSSAAFQVLQA   Vmp-3
119   DQK....IVQAMQEFKKSQKALGDLINSDDGQRVDNSNAKQSLQNNTVNNKSSIEQIIQA   Vmp-1
119   .........LEFAKFNEIKDKLQEYIKN..ELSKQEYEHIKQNIENELNKYTPIS.LEST   Lmp-1
108   .........EELNKLNKLRLDLQNLINSNDGQNVDSSDAKKALNENQIDDSLPIDKIKKT   Lmp-3

                     190        200        210        220        230        240
179   VPTIKKATKELQEAIKFKKQQEFNEFNKAKQELTDYIANDLNDLKYKDIKAKAEKSIEKV   Vmp-2
179   VQTIKKATKELREAIKFKKQQEFNEFNKIKQELTDYIANDLNDLKYKDIKAKAEKSIEKV   Vmp-3
175   ISKINEAKKELQSQINNARNQ.......................................   Vmp-1
167   LIEIQNATNNLIKLLNESTKEK......................................   Lmp-1
159   NENLENAKKELLNKINAERELQ......................................   Lmp-3

                     250        260        270        280        290        300
239   SDININSSFISDIQQATKLLIDAKKQAISEVEKINKSSELDKYKIAKNKLELLIKTEDAQ   Vmp-2
239   SDININSSFISDIQQATKLLIDAKKQAISEVEKINKSSELDKYKIAKNKLELLIKTEDAQ   Vmp-3
196   ............................................................   Vmp-1
189   ............................................................   Lmp-1
181   ............................................................   Lmp-3

                     310        320        330        340        350        360
299   MIKSANEKAKKILAENTTKDTTLSSEEFKTRRINLENALDELKEAIKNEKNIQKQEFEAK   Vmp-2
299   MIKSANEKAKKILAENTTKDTTLSSEEFKTRRINLENALDELKEAIKNEKNIQKQEFEAK   Vmp-3
196   ....................................................EKEVFEEK   Vmp-1
189   ....................................................DNIDNLNA   Lmp-1
181   ....................................................SKIFNE.K   Lmp-3
```

```
                    370                 380                 390                 400                 410                 420
359  KQELKILLESEWSKDIDKKNETAVWNNAKIDDSDSIKEIK.....EATKEVQGAIDSLIQ  Vmp-2
359  KQELKTLLESEWSKDIDKKNETTVWNNAKIDDSDSIKEIK.....EATKEVQGAIDSLIQ  Vmp-3
204  KQQLNKLIKSNEIDNSKKADETAILKNTNVVVGDSIKTIE.....TKTKEIEKAIESLTN  Vmp-1
197  KEQLKASISQANQLLPQLSDNDSEIAKAKKSLDAEIKNANQAVASNNTASMQSAKSSLDA  Lmp-1
188  KQELKRVLDLEDTKEVDFTKEQKVFIETNINETSSIEDIK.....NKIIEVEKATSSLTS  Lmp-3

                    430                 440                 450                 460                 470                 480
414  KMDEKNK......LNPLYAKEKFEKIQKQLQDLINSEDAKFIDTNKASEALKNTKITSTS  Vmp-2
414  KMDEKNK......LNPLYAKEKFEKIQKQLQDLINSEDAKFIDTNKASETLKNTKITSTS  Vmp-3
259  KINEFKKE.....QEKANVKAVFSKKSKQLKDLIDSEDGKKVDSSNESQVLTKTKIDENS  Vmp-1
257  KVAEITKKLETFNKDKEAKFNELKQTRNQIQEFINTNKNNPNYSELISQLTSKRDSKNSV  Lmp-1
243  KILNT..........KQQELQEFENIKKDLQDFINTKLNDAKYQSIKQKALDKINSLNGI  Lmp-3

                    490                 500                 510                 520                 530                 540
468  SIDEINQAIKTLKDAIVELKNAINGAKEDSIKEFDKVKKDLEVLVGSEDARSIEKRTEIG  Vmp-2
468  SIDEINQAIKTLKDAIVELKNAINGAKEDSIKEFDKVKKDLEVLVGSEDAKPIEKRTEIG  Vmp-3
314  SIEDIQNKTKDIEKAIESLTNKIN.............................DQKQQKN  Vmp-1
317  TDSSNKSDIESANTELKQALAKANADKVQ.....................ADNLAKSIKE  Lmp-1
293  NKNSTIKEIKAGGQNALIKAKEEAGLEKEK....................LD..GQNIKD  Lmp-3

                    550                 560                 570                 580                 590                 600
528  ILANTYILERDTIKQIKEKTEAIKKAISSLNKKIEDAKALKNELQKFNDIKAALEQLIKT  Vmp-2
528  ILANTYILERDTIKQIKEKTEAIKKAISSLNKKIANVKALKNELQKFNDIKAALEQLIKT  Vmp-3
345  MLNEVINKAKELVKKLVDSDSEIQQAKTQLDQEIQ.........................  Vmp-1
356  QLNNSVSNANTLSAKLTDKDNTIQQAKTELEKEVQ.........................  Lmp-1
330  TLKETINNAKEFKKLLIDNDQKIVDLKSNLDNEIS.........................  Lmp-3

                    610                 620                 630                 640                 650                 660
588  DDAKEVGTENASKALNNNKVNENSTLEEITKATKALEDAKSKLDQEIKAKKETEFNNLTV  Vmp-2
588  DDAKEVGTENASKALNNNKVNENSTLEEITKATKALEDAKSKLDQEIKTKKETEFNNLTV  Vmp-3
380  .KASQVVASNDTKAINSSKTSLDAKITDITKKLEAFN...........ATKKLEFTKLQE  Vmp-1
391  .KADQAIKSNNTASMQSAKSSLDAKVAEITKKLETFN...........KDKEAKFNELKQ  Lmp-1
365  .KAEQSLS.KDKESMESANDLLNTKLIEYKEILNKFN...........QEKEAKFNELEQ  Lmp-3

                    670                 680                 690                 700                 710                 720
648  EKGKLNELIAKSTDASAKALEEAKNVLAEVEKLNNSSPINLLKNAVIKTKDAIEKLNNNV  Vmp-2
648  EKGKLNELIAKSTDASAKAIEEAKKVLTEVEKLNNSSPINLLKNAVIKTKDAIEKLSNNV  Vmp-3
428  TRSNIDKFLTPEVKANP...........................................  Vmp-1
439  TRNQIQEFIN.TNKNNP...........................................  Lmp-1
412  TRKNIENFLTDEVKNNP...........................................  Lmp-3
```

```
                    730       740       750       760       770       780
 708  AQEKDKKAKLAEFNSIKTQLEELIAKDDAIQAGVNEAKKALQDNKADENSTLEEITKATK  Vmp-2
 708  AQEKDKKAKLAEFNSIKTQLEELIAKDDAIQAGVNEAKKALQDNKADENSTLEEITNATK  Vmp-3
 445  ..............................................NYTTLVNELEVAKK  Vmp-1
 455  ..............................................NYSELISQLTSKRD  Lmp-1
 429  ..............................................NYATLVKDLTNAKD  Lmp-3

                    790       800       810       820       830       840
 768  ALQEAK.AALELKISKAQEKAKQEFDTKKEQLKTLIGLSDANNVDKSNELNVLTNTSISK  Vmp-2
 768  ALQEAK.AALELKISKAQEKAKQEFDTKKEQLKTLIGLSDANNVDKSNELNVLTNTSISK  Vmp-3
 459  AKEVSE.SSNKSDIVAANNELKQAFQTAQSSKNDADKTSNEEKAKLSASLSNAKKLDKNL  Vmp-1
 469  SKNSVTDSSNKSDIESANTELKQALAKANADKVQADNLAKSIKEQLNNSVSNANTLSAKL  Lmp-1
 443  AKKSVTNSSNKSDIIAANEALIQALADANKAKDQVDEANKSIKEQLNALIDKANTLLPQL  Lmp-3

                    850       860       870       880       890       900
 827  TDSIKEIKQKTSTIEEAIKTLNKKVQDKKNEKIQEFEKAKESLEKLVKEE.DAKEVGIDS  Vmp-2
 827  TDSIKEIKQKTSTIEEAIKTLNKKVQDKKNEKFREFEKAKESLKKLVKRRRCKKKLALIV  Vmp-3
 518  TDSDGEIQQAKAELAEEVEKANQAITSNN...............................  Vmp-1
 529  TDKDNTIQQAKTELEKEVQKADQAIKSNN...............................  Lmp-1
 503  NDNDSEIVKAKESLNAEITNANKAVNQND...............................  Lmp-3

                    910       920       930       940       950       960
 886  ANEALKTKVDEKLTIEEIKNAILKISNEETKLKSSIESAKHQAKETFDSKKQELKSLLDS  Vmp-2
 887  QTKLLKTKVDEKLTIEEIKNAILKISNEETKLKSSIESAKQQAKETFNSKKQELKSLLDF  Vmp-3
 547  TKEIQNSNTSLLNKISEVKNKLDKFSNEKEAEFNKLEASRSAIKEFIN............  Vmp-1
 558  TASMQSAKSWLDAKVAEITKKLETFNKDKEAKFNELKQTRNQIQEFIN............  Lmp-1
 532  NASMQSAKSSLDDKVTKIQNQLTEFNKDKDAKFKELEQTRKDIDNFLT............  Lmp-3

                    970       980       990      1000      1010      1020
 946  TTSEVDKKAEEEKYNKTTIDNDSTIKQIKQKTSDIENAITTLKEKIAKVKGDKAVELQKF  Vmp-2
 947  TTSEVDKKAEEEKYNKTTIDNDSTIKQIKQKTSDIENAISTLKEKIAKAKGDKAVELQKF  Vmp-3
 595  ..............ENNTNPNYTALIQKLQAKLDAKNSITKSSNKSDIIATN........  Vmp-1
 606  .............TN.KNNPNYSELISQLTSKRDSKNSVTDSSNKSDIESAN........  Lmp-1
 580  .............DDVKNNPNYATLVKDLTNAKDDKKSVTKSSNKSEIIAAN........  Lmp-3

                   1030      1040      1050      1060      1070      1080
1006  NLVKQQLEELIAKEVAKEVGTSKAEEALKVNVVDQNSTLDAISKATKALGDAKSEFAQNI  Vmp-2
1007  NLVKQQLEELIAKEDAKEVGTSKAEEALKVNVADENSTLDAISKATKALEDVKAELTQNI  Vmp-3
 633  ....QALQEALAIEKTEKESANSQNQAVKN...TLNETIGKAKELDKNLTDSDGEIQQAK  Vmp-1
 644  ....TELKQALAKANADKVQADNLAKSIKEQ...LNNSVSNANTLSAKLTDKDNTIQQAK  Lmp-1
 619  ....DELKQALDKAKVAKDQIDEANKSIKEQ...LSDSITNANQLLNKLVDSDKDIQKAK  Lmp-3
```

```
               1090      1100      1110      1120      1130      1140
1066    IDTKNNATKTFNDKKDELNKLLDTSDAKSVDNKKESDVLKDNSIDSNTPIKEIKAKTEKI  Vmp-2
1067    TDAKDNATKTFNDKKDELKKLLESSDAKIVDNKKESDVLNGNSVDSNTPIKEIKAKTEKI  Vmp-3
 686    AELTNEIEKANQTIASNNTALMENSNTSLLN...........................KI  Vmp-1
 697    TELEKEVQKANQAIKSNNTASMQSAKSSLD...........................AKV  Lmp-1
 672    TELSQEIQSASQELNLNNPTSMQSAKESLD...........................AKV  Lmp-3

               1150      1160      1170      1180      1190      1200
1126    TEAINSLTTSIKNKKDEEFNKYNDIKTSLENLIKEEDAVQVGIADVQKTLSENNVDKTAT  Vmp-2
1127    TEAINSLTTSINNKKDDEFNKYNAIKTSLENLIKEEDAVQVGIADVQKTLSENDVDKTAT  Vmp-3
 719    SEVQNKLDKFNNDKK.AEFNKLQELKNKIDDFEKK.........................  Vmp-1
 730    AEITKKLETFNKDK.EAKFNELKQTRNQIQEFIN..........................  Lmp-1
 705    TEITKKLETFNKDK.DVKFKELEKTRKDIDEFIN..........................  Lmp-3

               1210      1220      1230      1240      1250      1260
1186    IEKIQHSTEALTHAKEELKKLIDTTKKQLTKEFETKKSELEKLISLPDANNVDKKDELSI  Vmp-2
1187    IEKIQHSTEALTHVKEELQRLIDNTKKQLTKEFENKKSELEKLISLPDANNVDKKDELSI  Vmp-3
 753    ..................................NKNNEIYSKFNLDELINKSVQIKNSL  Vmp-1
 763    .................................TNKNNPNYSELISQLTSKRDSKN....  Lmp-1
 738    .................................TNKTNPNYSTLISELTSKRDSKN....  Lmp-3

               1270      1280      1290      1300      1310      1320
1246    FGNTNITNSDSIKQIKEKITKIQNALESLTNKISKQK...EQELEKYNTTKTALEQLIKD  Vmp-2
1247    FGNINITNSDSIKQIKEKITKIQNALKSLTDKISKQK...EQELEKYNTTNTALEQLIKD  Vmp-3
 779    G...SINESSNKKDIVDANKKMQDALNELQAKMAEIH...KKTFQEFNEHKNELENLIKK  Vmp-1
 786    ....SVTDSSNKSDIESANTELKQALAKANADKVQADNLAKSIKEQLNNSVSNANTLSAK  Lmp-1
 761    ....SVTNSSNKSDIETANTELKQALAKANTDKAQADNLAKSTKEQLNNSISSANTLLAK  Lmp-3

               1330      1340      1350      1360      1370      1380
1303    EDAKEVGTTDANAAITKNKADKNSTLEEITNATKALEDAKSKLDQEIKTKKEAEFNNLTK  Vmp-2
1304    EDAKEVDTTEATTALTKTKADKNSTLEEIANATKALEDAKSKLDQEIKTKKEAEFNNLTN  Vmp-3
 833    EDAKEVGTDEANTAITNNDVKENSSIEEITKATKALDEAKSKLDQKINTQKATELNSLNE  Vmp-1
 842    LTDKDNTIQQAKTELEKEVQKANQAIKSNN..TASMQSAKSSLDAKVAEITKKLETFNKD  Lmp-1
 817    LTDKDNTIQQAKTELEKEVQKANQAVASNN..TASMQSAKSSLDAKVTEITKKLETFNKD  Lmp-3

               1390      1400      1410      1420      1430      1440
1363    AKTELSDLITSSSNQAPADAISDAQKTLDEINKLNLTNVSTIKSMKDATQK..IEAANKA  Vmp-2
1364    AKTELSNLITSSSNQAPDEAISDAQKTLDEINKLNLTNVSTIKSMKDATQK..IKDANEA  Vmp-3
 893    SKEKLNNLITSSSNQVSAEEISKAKKVLEEINNLSLNNDSSIKSLKEATQK..IKDAETQ  Vmp-1
 900    KEAKFNELKQTRNQIQEFINTNKNNPNYSELISQLTSKRDSKNSVTDSSNKSDIESANTE  Lmp-1
 875    KDVKFKELEQTRKDIDEFINTNKTNPDYSTLISELTSKRDSKNSITNSSNKSDIETANTE  Lmp-3
```

```
                      1450        1460        1470        1480        1490        1500
1421  LKQAIEKLKAEK...LQKFNEAKTALENLVKEEDAIQTGVEAAKKALDDNNKINKNSTLE  Vmp-2
1422  LKQAIEKLEAEKTEKLQKFNEAKNALENLVKEEDAIQTGVEAAKKALDDNNKINKNSTLE  Vmp-3
 951  LTKEIEKAKIEKTDKLKKFNEVKKSLEDLIKDDDAIQVGTDDAPKLLEDNNNINENSSIE  Vmp-1
 960  LKQALAKANADKVQADNLAKSIKEQLNNSVSNANTLSAKLTDK....DNTIQQAKTELEK  Lmp-1
 935  LKQALAKANTDKDQADNLARSTKEQLNKSISSANTLLAKLTDK....DNTIQQAKTELEK  Lmp-3

                      1510        1520        1530        1540        1550        1560
1478  EITNATKALEDAKSKLDQEIKTKKEAEFNNLTNAKTELSNLITKQAPAEAISKAKKVLEE  Vmp-2
1482  EITSATKALEDAKSKLDQEIKAKKEAEFNNLTNAKTELSDLITKQAPAEAISNAKKVLEE  Vmp-3
1011  EIINATKTLEDGKSKLDKKIKTKKQPLIRDLKRKVDDLSRWLEYSSNSEEHFAALDPDKR  Vmp-1
1016  EVQKANQAIKSNNTASMQSAKSSLDAKVAEITKKLETFN..KDKEAKFNELKQTRNQIQE  Lmp-1
 991  EVQKANQAVASNNTASMQSAKSSLDAKVTEITKKLETFN..KDKDVKFRELEQTRKDIDE  Lmp-3

                      1570        1580        1590        1600        1610        1620
1538  INGLNLSNDSSIKSLKDATQKIKDAETQLTAEI...........................  Vmp-2
1542  INGLSXSNDSSIKSLKDATQKIKDAETQLTKEIKKLKAEKTEKLQKFNEAKTALENLVKE  Vmp-3
1071  PK..NITLDTIKKDLEDARRLLQEANKLN...............................  Vmp-1
1074  FINTNKNNPNYSELISQLTSKRDSKNSVT...............................  Lmp-1
1049  FINTNKTNPNYSTLISELTSKRDSKNSIT...............................  Lmp-3

                      1630        1640        1650        1660        1670        1680
1571  ............................................................  Vmp-2
1602  EDAIQTGVEAAKKVLEDNNANENSTLDEITKATKALEDAKSKLDQEIKTKKEAEFNNLTN  Vmp-3
1098  ............................................................  Vmp-1
1103  ............................................................  Lmp-1
1078  ............................................................  Lmp-3

                      1690        1700        1710        1720        1730        1740
1571  ............................................................  Vmp-2
1662  AKTELSNLIASNNKQAPADAISAAQKTLDEINKLNLTNVSTIKSLKDATQKIKDANEALK  Vmp-3
1098  ............................................................  Vmp-1
1103  ............................................................  Lmp-1
1078  ............................................................  Lmp-3

                      1750        1760        1770        1780        1790        1800
1571  ........EKTEKLKEFDEARKSLRELIKDDDAKEVETSKAKEALKTN.K...ADENSSI  Vmp-2
1722  QAIEKLEAEKTEKLQKFNEAKKALEDLIKDEDAKEVGTNDAQKLLEDNNK...INKNSSI  Vmp-3
1098  .........DDSKIANINKKIDEIKELNKKTNLVFAIEYAFEYLARKIEN...SEKSIDY  Vmp-1
1103  .........DSSNKSDIESANTELKQALAKANADKVQADNLAKSIKEQLNNSVSNANTLS  Lmp-1
1078  .........NSSNKSDIETANTELKQALAKANTDKAQADNLARSTKEQLNKSISSANTLL  Lmp-3
```

```
                1810      1820      1830      1840      1850      1860
1619  EEIIKATKALEDAKFELNQEIRVQKDRLMDKLNAKNKKLDALLKTENLDDLMSIEKLIG.  Vmp-2
1779  DEITNATKALDEAKSKLGQKIKAKKQELMASLKEKNEKLDTLLKTENLDDLMVISKRPDD  Vmp-3
1146  LKEFKSKEKNREFVTKLREKVEKIKKLSKENKIPNLKKAAEYLENLAIIPLLLFQYNFYY  Vmp-1
1154  AKLTDKDNTIQQAKTELEKEVQKANQAIKSNNTASMQSAKSSLDAK....VAEITKKLET  Lmp-1
1129  AKLTDKDNTIQQAKTELEKEVQKANQAVASNNTVSMQSAKSSLDTK....VTEITKKLET  Lmp-3

                1870      1880      1890      1900      1910      1920
1678  LSAGEIKNKLEDAKKLLQEAKGLSENSKITNIKQKIQKIDEYVRQFEPWISNMLKIKGGL  Vmp-2
1839  LNADEIKNKLEKARKLSQEARDLSENSKITDINQKIQKIDEFVGKLESWIKVMEEEKKPI  Vmp-3
1206  ELPATLQEKLIKEFENILDKETIPSDSEITELENKSIELMIAQDPKIAFTILQKQSKD..  Vmp-1
1210  FNKDKEAKFNELKQTRNQIQEFINTNKNNPNYSELISQLTSKRDSKNSVTDSSNKSD...  Lmp-1
1185  FNKDKEAKFNELKKTRGQIQEFINTNKNNPNYSELISQLTSKRDSKNSVTNSSNKSD...  Lmp-3

                1930      1940      1950      1960      1970      1980
1738  VSVINSNIEQLKKFKAKKFNNNFISKLNEKFHSNIFNNLSKNMKLADLKKIEEDSVNLLR  Vmp-2
1899  AHRIKFRIENLKQIKANEFYSVFLDKFKRVLEKPIFNNPSWTKKLSDLKKADEDSDNLWK  Vmp-3
1264  ...LSFELRNFIELHDNGYNNFRTMNNSLKTIEDKYNLALEKNDLDALKKEIIKMENFYK  Vmp-1
1267  ...IESANTELKQALAKANADKVQADNLAKSIKEQLNNSVSNANTLSAKLTDKDNTIQQA  Lmp-1
1242  ...IETANTELKQALAKANTDKAQADNLARSTKEQLNKSISSANTLLAKLTDKDNTIQQA  Lmp-3

                1990      2000      2010      2020      2030      2040
1798  RVKLQEKYENDDKLSPSLQKKTCKRIWKNIR                               Vmp-2
1959  LCQLFSQYKNDTNLSPALQKKLAKEFEKILAKETIPSDSEITELENKLKKIVEEDPTTEF  Vmp-3
1321  LIKTFHLYLCEIGFIKSMFYYDDKKEEVSKQAFECAKNALNDLAS...............  Vmp-1
1324  KTELEKEVQKANQAIKSNNTA                                         Lmp-1
1299  KTEL                                                          Lmp-3

                2050      2060      2070      2080      2090      2100
1829                                                                Vmp-2
2019  SNLQNEAKDTKKLIENLVPESAKELDGFKKLQSWINNALKLIEEGYNSALQKEDLDALKV  Vmp-3
1366  ............................................................  Vmp-1
1345                                                                Lmp-1
1303                                                                Lmp-3

                2110      2120      2130      2140      2150      2160
1829                                                                Vmp-2
2079  ETSKINNLNELIKLFYTYWKQFNYLISDKHDQREVFKRGIKDADEAINALGQINLNQDLG  Vmp-3
1366  ........................................................QDIS  Vmp-1
1345                                                                Lmp-1
1303                                                                Lmp-3
```

```
                 2170           2180           2190
                   .              .              .
1829                                                  Vmp-2
2139  NDKLMKEKIKEIENELATAQSLTESHFKH                   Vmp-3
1370  NKELISKKVTDIKNEFYKAYINVRKFCLND                  Vmp-1
1345                                                  Lmp-1
1303                                                  Lmp-3
```

X non conserved
X similar
X conserved
X all match
